# Identifying patients who will not reachieve remission after breakthrough seizures

**DOI:** 10.1111/epi.14697

**Published:** 2019-03-22

**Authors:** David M. Hughes, Laura J. Bonnett, Anthony G. Marson, Marta García‐Fiñana

**Affiliations:** ^1^ Department of Biostatistics Institute of Translational Medicine University of Liverpool Liverpool UK; ^2^ Department of Molecular and Clinical Pharmacology Institute of Translational Medicine University of Liverpool Liverpool UK; ^3^ The Walton Centre NHS Foundation Trust members of Liverpool Health Partners Liverpool UK

**Keywords:** breakthrough, dynamic classification, epilepsy, focal, generalized, remission

## Abstract

**Objective:**

We aim to identify people with epilepsy who are unlikely to reachieve a 12‐month remission within 2 years after experiencing a breakthrough seizure following an initial 12‐month remission.

**Methods:**

We apply a novel longitudinal discriminant approach to data from the Standard and New Antiepileptic Drugs study to dynamically predict the risk of a patient not achieving a second remission after a breakthrough seizure by combining both baseline covariates (collected at the time of breakthrough seizure) and follow‐up data.

**Results:**

The model classifies 83% of patients. Of these, 73% of patients (95% confidence interval [CI] = 58%‐88%) who did not achieve a second remission were correctly identified (sensitivity), and 84% of patients (95% CI = 69%‐96%) who achieved a second remission were correctly identified (specificity). The area under the curve from our model was 87% (95% CI = 80%‐94%). Patients who did not achieve a second remission were correctly identified on average after 10 months of observation postbreakthrough. Occurrence of seizures after breakthrough and the number of seizures experienced were the most informative longitudinal variables. These longitudinal profiles were influenced by the following baseline covariates: age at breakthrough seizure, presence of neurological insult, and number of antiepileptic drugs required to achieve first remission.

**Significance:**

Using longitudinal data gathered during patient follow‐up allows more accurate predictions than using baseline covariates in a standard Cox model. The model developed in this paper is a useful first step in developing a tool for identifying patients who develop drug resistance after an initial remission.

**Key Points**

- The presence and number of post–breakthrough seizures are the most informative variables for predicting poor outcome following first breakthrough 
- Age at breakthrough seizure, neurological insult, and number of treatments required to achieve first 12‐month remission are also influential
- Real‐time monitoring of seizure history leads to more accurate predictions than those estimated from existing models at baseline, with approximately 10 months of observation required on average to correctly identify patients who do not reachieve remission

## 1 INTRODUCTION

Approximately 30%‐40% of patients with epilepsy will never enter a sustained remission from seizures despite multiple treatment changes.^1^ Of the 60%‐70% of patients who achieve a 12‐month remission from seizures, around 37% may have a breakthrough seizure, defined here as one that occurs following a remission of at least 12 months, despite continued treatment with antiepileptic drugs (AEDs).^2^

Following a breakthrough, seizure patients may go on to achieve a further period of remission, either immediately or following changes to their treatment regimen.^3^ However, some continue to experience seizures despite multiple treatment changes.

If we were able to reliably predict prognosis following a breakthrough seizure, patients could be provided with important information that could influence life choices, and medical resources could be used more effectively; for example, patients could be put on a pathway for respective surgery earlier in their disease course.

However, very little work has been conducted to investigate patient outcomes following breakthrough seizures. Bonnett et al^4^, ^5^ considered prognostic factors for the risk of a breakthrough seizure following a period of remission, the risk of seizure recurrence following a breakthrough seizure, and the chance of achieving a 12‐month period of remission following a breakthrough seizure. These studies considered patient data up until the breakthrough seizure as prognostic variables, but not data collected during subsequent follow‐up. In addition, one study considered prognostic factors that affect seizure relapse and the development of drug resistance in patients who had experienced long‐term remission.^6^ Two further studies investigated breakthrough seizures in Uganda^7^ and Egypt,^8^ although neither study considered outcomes following the breakthrough seizure.

A few studies have attempted to predict patients' long‐term epilepsy status with various length of observation.^9^, ^10^, ^11^ Hughes et al^12^ described a model that identifies patients who will not achieve a 12‐month continuous seizure‐free period within 5 years of initial diagnosis. However, this study only considered patients up until their first remission if a remission was observed. Keller et al^13^ identified patients who will continue to experience seizures following brain surgery. To the best of our knowledge, no models exist that aim to dynamically identify patients who will not achieve remission following a breakthrough seizure.

Our aim in this study was to describe in a statistical model the seizure history of patients following a breakthrough seizure and use the model to give quantitative predictions. Therefore, further evidence could be added to support clinician intuition regarding the trajectory of patients not likely to achieve a second remission after a breakthrough seizure.

## 2 MATERIALS AND METHODS

The Standard and New Antiepileptic Drugs (SANAD) trial has been described in detail elsewhere.^14^, ^15^ In summary, the trial was designed to compare two standard AEDs with a range of alternatives. Patients were eligible for the trial if they were at least 5 years old and had experienced at least two clinically definite unprovoked seizures in the past year. Patients for whom carbamazepine was considered to be the standard optimal treatment were recruited to Arm A of the SANAD trial and were randomly allocated in equal proportions to receive either carbamazepine, gabapentin, lamotrigine, or topiramate. From June 1, 2001, a further drug, oxcarbazepine, was added to the trial, and patients were allocated to these five drugs in equal proportions. Patients for whom valproate was considered the standard optimal treatment were included in Arm B of the trial and were randomly assigned in equal proportions to valproate, lamotrigine, or topiramate.

The SANAD study has previously been used to investigate time to treatment failure from randomization and time to 12‐month remission from randomization.^16^, ^17^, ^18^

SANAD is the largest prospective study in patients with epilepsy to date and contains follow‐up data for up to 7 years, allowing an excellent opportunity to investigate time‐dependent factors that influence the risk of having drug‐resistant epilepsy.

This analysis describes a model that combines both baseline covariates (recorded at the time of breakthrough seizure) and subsequent follow‐up data to identify patients who will not achieve a second period of 12‐month remission within 2 years following a breakthrough seizure. A patient's classification is determined by the likelihood that the patient will achieve a second remission, and the estimation of this probability can be updated with new data while the patient remains under observation and is not classified as achieving a second remission.

This analysis considers both arms of the SANAD study simultaneously. Because the allocation to Arm A or B was dependent on the type of epilepsy, we consider type of epilepsy as a potential baseline covariate. Patients who experienced a 12‐month continuous seizure‐free period since randomization and then subsequently had a breakthrough seizure were eligible for inclusion in this analysis.

### 2.1 Statistical analysis

The primary outcome of interest is whether a patient experiences a further 12‐month remission within 2 years of experiencing a breakthrough seizure.

We considered four variables that were recorded at each follow‐up visit and modeled the changes in these variables over time using a multivariate generalized linear mixed model.^19^ Separate models were fitted to patients who are known to have achieved another remission period of 12 months within 2 years following breakthrough and those who did not. Specifically we consider (1) whether a patient had experienced seizures since their previous clinic visit (yes/no), (2) how many seizures were experienced since the previous clinic visit, (3) the number of patient‐reported adverse events experienced since the previous clinic visit, and (4) whether a patient's treatment was changed at the last clinic visit (yes/no). Treatment change included changes in dose (increase or decrease) of a drug or the addition or removal of a drug. Potential adverse events included depression, dizziness, allergic reactions, headaches, and tiredness among others.^20^

Each variable that was measured at repeated clinic visits (longitudinal variable) was allowed to depend upon the following baseline variables: age at breakthrough seizure, sex, electroencephalogram result at randomization, computerized tomography or magnetic resonance imaging result at randomization, type of epilepsy, first degree relative with epilepsy, neurological insult (learning difficulties or a neurological deficit), total number of seizures prior to first remission, total number of treatments prior to first remission, time to first period of 12‐month remission, number of tonic–clonic seizures prior to breakthrough seizure, and post–breakthrough seizure treatment decision.^4^, ^5^ In addition, time since the last clinic visit was included to account for clinic visits not being equally spaced. Baseline covariates from the above list were included in a forward selection approach with models compared using penalized expected deviance.^21^ The best combination of baseline covariates to explain each longitudinal variable was determined.

These two fitted models were subsequently used in a longitudinal discriminant analysis^22^ to classify patients as either likely to achieve another period of 12‐month remission within 2 years of breakthrough seizure or likely to not reachieve 12‐month remission within 2 years. A longitudinal discriminant analysis assesses the likelihood that the data of a new patient were generated by each of the two multivariate mixed models. In this sense, the model assesses in a probabilistic manner which of the two average group trends the new patient is closest to.

At each follow‐up visit for a patient, between their initial breakthrough seizure and their 2‐year post–breakthrough endpoint, their risk of not achieving remission within 2 years of breakthrough seizure was updated using the additional information collected at the visit. The best combination of the four longitudinal variables to be used to classify new patients was selected using the probability of correct classification (PCC), to maximize classification accuracy.

**Figure 1:**
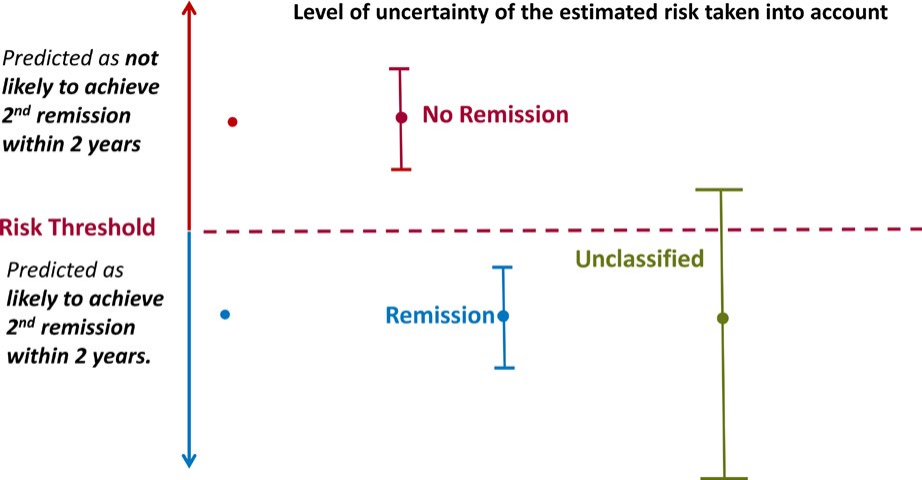
Allocation scheme for identifying patients who will not reachieve remission within 2 years of breakthrough seizure

Credible intervals around the calculated probability of not achieving remission within 2 years of breakthrough were used to assess the precision of the estimated probability. At each follow‐up visit, if the calculated credible interval was entirely above a threshold of 0.38 (threshold determined following a receiver operating characteristic [ROC] curve analysis as the point on the ROC curve closest to the top‐left corner of the plot), then the patient was classified as not going to achieve remission within the remaining period of the 2 years since their breakthrough seizure. If this was not the case, then the patient simply remained under observation. At the final follow‐up visit before 2‐year status was confirmed, there was an additional option of classifying a patient as likely to achieve remission if the credible interval was entirely below 0.38. If the credible interval contained the threshold at this visit, then the patient remained unclassified, as there was insufficient confidence to predict the patient's status. Figure 1 gives a diagrammatic representation of this classification scheme.

Patients were predicted as likely not to achieve remission only at the point in follow‐up at which their credible interval for risk of drug resistance is entirely above 0.38. This means that not all patients are classified at the same time and classification only occurs when there is reasonable confidence that a patient will truly not achieve remission within 2 years of breakthrough.

To test the predictive accuracy of our model, data from 70% of the patients in each group (those who achieved a second remission and those who did not) were used to train the model, and the remaining 30% were used to test the predictive accuracy. This was repeated for 100 random splits of the data into training and test sets. Predictive accuracy measures were then calculated and averaged.

For comparison purposes, we compared our longitudinal model with a Cox proportional hazards model described in Bonnett et al.^23^ The Cox model predicts at baseline (time of breakthrough seizure) the probability of not achieving remission within 2 years.

## 3 RESULTS

Of 2437 patients who were considered for this analysis, 1901 patients were excluded for a number of reasons; in 58 patients, the seizures were later linked to causes unrelated to epilepsy (3%), 786 patients did not achieve remission during the follow‐up period (41%), and 1057 patients did not experience a breakthrough seizure (56%). In total, 536 patients experienced a breakthrough seizure (34% of all patients who experienced remission). Patients who had a dose decrease prior to a breakthrough seizure were also excluded from this analysis (n = 26, 4.9% of all patients who experienced a breakthrough seizure), because their seizure could potentially be due to AED withdrawal.^4^, ^5^ A further 210 patients were excluded because, although they did experience a breakthrough seizure, they were not followed for sufficient time to determine their 2‐year status postbreakthrough. Of the remaining 300 patients who experienced a breakthrough seizure, 185 patients (62%) went on to achieve a further period of 12‐month seizure remission within 2 years of experiencing their breakthrough seizure and 115 patients (38%) were observed for 2 years following breakthrough seizure without experiencing a 12‐month remission.

Table 1 describes the characteristics of patients who were observed to achieve a second period of 12‐month remission within 2 years of breakthrough and those who did not. The best combination of longitudinal variables to achieve optimal classification accuracy was a bivariate model including whether the patient experienced seizures since their last visit and the total number of seizures experienced since the patient's last visit. Separate bivariate models were fit to the patients who were observed to achieve remission and those who did not. The two bivariate models were used in the longitudinal discriminant analysis.

**Table 1:** Patient demographics for patients who have and have not experienced a breakthrough seizure and have been observed for long enough to determine their 2‐year status

Characteristic	Patients who achieve 12‐mo remission within 2 y of breakthrough seizure	Patients who do not achieve 12‐mo remission within 2 y of breakthrough seizure
	Total, n = 185	Total, n = 115
Male	114 (62%)	61 (53%)
Epilepsy in first degree relative	26 (14%)	15 (13%)
Neurological insult	31 (17%)	19 (17%)
Epilepsy type		
Focal	105 (57%)	80 (70%)
Generalized	51 (28%)	23 (20%)
Unclassified	29 (16%)	12 (10%)
EEG results		
Normal	58 (32%)	49 (43%)
Abnormal	110 (59%)	61 (53%)
Not done	17 (9%)	5 (4%)
CT/MRI scan results		
Normal	91 (49%)	59 (52%)
Abnormal	33 (18%)	28 (24%)
Not done	61 (33%)	28 (24%)
Drugs attempted to achieve 12‐mo remission		
One	135 (73%)	79 (69%)
Two or more	50 (27%)	36 (31%)
Number of tonic–clonic seizures ever until first breakthrough seizure, median (IQR)	2 (1‐5)	2 (0‐6)
Total number of seizures before diagnosis, median (IQR)	10 (3‐51)	20 (5‐100)
Age at first breakthrough seizure, median (IQR)	24 (16‐44)	35 (20‐50)
Time to achieve 12‐mo remission from randomization, y, median (IQR)	1 (1‐1.52)	1.24 (1.0‐2.0)
Breakthrough seizure treatment decision		
No change to treatment plan	123 (66%)	60 (52%)
Increased dosage	59 (32%)	52 (45%)
Decreased dosage or not specified	3 (2%)	3 (3%)

CT, computed tomography; EEG, electroencephalogram; IQR, interquartile range; MRI, magnetic resonance imaging.

The covariates used to model each longitudinal variable are shown in Table 2. For both groups of patients (those who achieved remission and those who did not), the likelihood of experiencing seizures decreased (odds ratios < 1) as time since breakthrough increased. Conversely, as the time since last follow‐up increased, the likelihood of experiencing seizures increased (odds ratios > 1), probably reflecting that they had a longer period in which to experience a seizure. Time since breakthrough and since last follow‐up was also associated with the expected number of seizures but with a minor effect. Patients who ultimately achieved second remission, but had required more AEDs to achieve their first period of 12‐month remission, were expected to experience slightly more seizures than similar patients requiring fewer AEDs (parameter estimate = 0.207, 95% confidence interval [CI] = 0.116‐0.302, implying that for each drug required to achieve a patient's first 12‐month remission, the number of seizures they were expected to have experienced since their last, postbreakthrough follow‐up visit increased by 0.207). Patients who had neurological insult on diagnosis, but ultimately achieved remission, were approximately four times more likely to experience seizures than those who did not have neurological insult. For patients who would not achieve a second remission, increasing age increased the risk of experiencing seizures, although the result was not statistically significant.

**Table 2:** Model fixed‐effects parameters for the multivariate mixed model

Patient group	Variable	Longitudinal variable			
		Seizures since last visit, yes/no		Total number of seizures since last visit	
		Odds ratio^a^	95% CI	Parameter estimate^a^	95% CI
Patients who achieve second remission	Time since last follow‐up, mo	2.657	2.002 to 3.495	0.024	0.012 to 0.036
	Time since breakthrough, mo	0.426	0.330 to 0.543	−0.069	−0.080 to −0.057
	Drugs attempted to achieve first remission			0.207	0.116 to 0.302
	Age at breakthrough, y			0.002	−0.001 to 0.005
	Neurological insult	3.739	1.147 to 11.928		
Patients with no second remission observed	Time since last follow‐up, mo	1.487	1.291 to 1.730	0.045	0.021 to 0.070
	Time since breakthrough, mo	0.819	0.769 to 0.872	−0.021	−0.039 to −0.030
	Age at breakthrough, y	1.030	1.010 to 1.053	0.006	−0.004 to 0.014

Blank entries show that the variable was not included in the submodel for the longitudinal variable described in that column. CI, confidence interval.

Odds ratios represent the predicted increase/reduction in the odds of experiencing seizures for a given covariate (per one unit increase in continuous covariates, or due to the presence of a binary covariate).

a Parameter estimates relate to the predicted increase/reduction of the total number of seizures for a given covariate (per one unit increase in continuous covariates, or due to the presence of a binary covariate).

**Figure 2:**
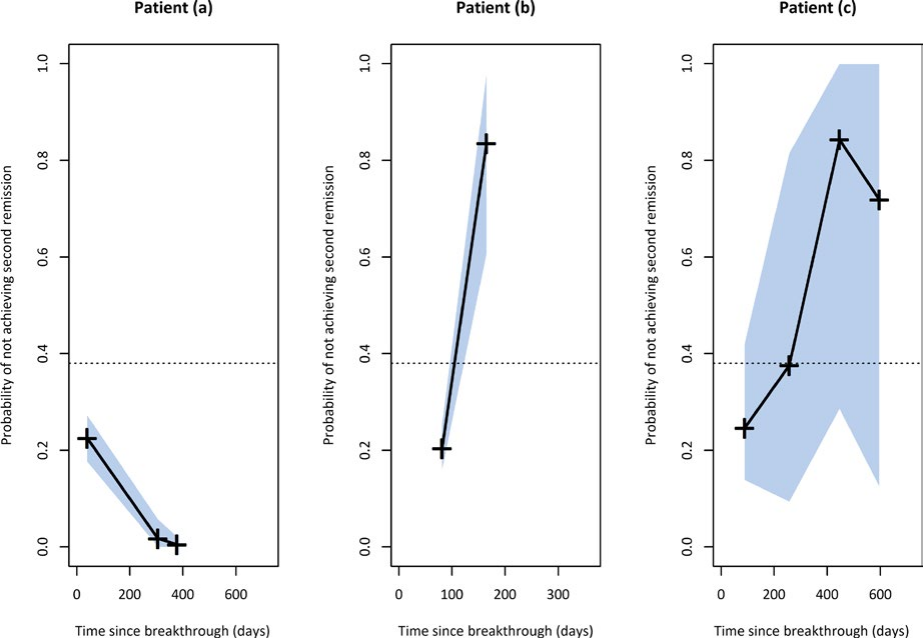
Three individual patients*'* probabilities of not achieving a second remission. The crosses show the probability assigned by the model at the clinical visits, and the gray shaded areas represent 99% credible bands around the predicted probabilities. The dotted line denotes the threshold of 0.38 used in the classification scheme

To demonstrate how our model works, we describe the clinical follow‐up postbreakthrough of three patients (Figure 2). Patient (a) was correctly identified to achieve a second remission. At their first visit, 39 days postbreakthrough, they reported having experienced two seizures, giving a probability of not achieving remission of 0.23 at this visit. However, at two subsequent visits they reported no seizures, and accordingly the model gives a low probability that they will not achieve a second remission. This was confirmed at their next visit, when it was observed that they had been seizure‐free for 12 months.

Patient (b) was correctly identified by the model as not likely to achieve a second period of 12‐month remission following a breakthrough seizure. At their first two visits postbreakthrough (81 and 165 days), they described experiencing two and 10 seizures since the previous visit, respectively. At the second visit, the model assigned a high probability of not achieving a second remission to this patient. In addition, the credible interval around this probability was entirely above the threshold of 0.38, and the patient was classified as not going to achieve remission within 2 years of breakthrough. This prediction was made at 165 days postbreakthrough.

Patient (c) was unclassified by our model despite ultimately being observed to achieve remission, because the credible intervals were too wide to determine with confidence the patient's status (gray shaded area).

The predictive accuracy of our model is assessed by considering how many of the patients were correctly classified (Table 3). Of the patients classified by the model, 73% of patients (95% CI = 58%‐88%) who would not achieve a second remission were correctly identified (sensitivity), and 84% of patients (95% CI = 69%‐96%) who achieved a second remission were correctly identified (specificity). Overall, 80% of patients (95% CI = 71%‐89%) were correctly identified (PCC). The area under curve (AUC) from our model was 87% (95% CI = 80%‐94%), showing that the model achieves a good level of discrimination. Of the patients predicted not to achieve remission, 73% of patients (95% CI = 57%‐91%) were observed not to achieve remission (positive predictive value), and 85% (95% CI = 77%‐93%) of patients predicted to achieve remission went on to achieve remission (negative predictive value). The prediction times reported in Table 3 show the average time at which a patient is correctly identified as not going to achieve remission from seizures, and show that our model is able to identify patients who will not achieve a second remission approximately 10 months after a breakthrough on average.

**Table 3:** Prediction accuracy of the discriminant analysis models and predictions at baseline from a Cox proportional hazards model

	Longitudinal discriminant analysis	Cox model
Optimal cutoff	0.38	0.37
Sensitivity	0.73	0.62
Specificity	0.84	0.66
PCC	0.80	0.64
AUC	0.87	0.66
PPV	0.73	0.53
NPV	0.85	0.74
Unclassified, %	17	0
Mean lead time, d	372	730
Mean prediction time, d	303	0

The accuracies recorded are the averages across 100 splits of the data into training and test sets.

AUC, area under curve; NPV, negative predictive value; PCC, probability of correct classification; PPV, positive predictive value.

The predictive accuracies reported above are based on patients who were classified by the model. Approximately 17% of patients (95% CI = 16%‐18%) were left unclassified by our model, as there was considerable uncertainty about their status and longer follow‐up would have been required. If unclassified patients were considered as incorrectly classified, the predictive accuracy would indicate a sensitivity of 57% (95% CI = 41%‐71%), specificity of 72% (95% CI = 59%‐87%), and PCC of 66% (95% CI = 57%‐74%). A key point of this approach is that by leaving a relatively small proportion of patients unclassified, much greater predictive accuracy is obtained for patients who are classified.

**Figure 3:**
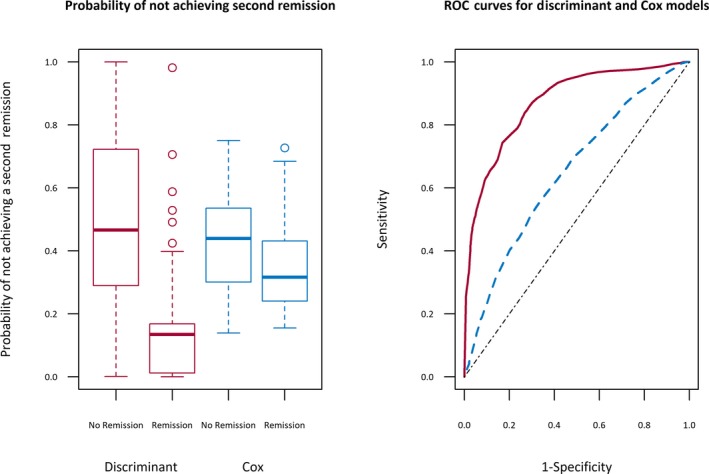
Left panel, Box plots showing the probabilities assigned to patients who achieved a second remission and those who did not for both the discriminant model and the Cox model. Right panel, Corresponding receiver operating characteristic (ROC) plot for the discriminant model (solid red curve) and the Cox model (dashed blue curve)

For comparison purposes, we compared our model predictions to predictions from the Cox proportional hazards model described in Bonnett et al.^23^ The predictive accuracy of the Cox model (used to predict chance of experiencing remission within 2 years) is also shown in Table 3, and the corresponding ROC curves are shown in Figure 3. The longitudinal model achieves substantially better classification accuracy. This is emphasized by the box plots in the left panel of Figure 3, which show much greater separation in the probabilities assigned to patients in each group for the discriminant model than the Cox proportional hazards model. This demonstrates that the information available at the point of breakthrough seizure is insufficient to determine whether a patient will go on to achieve remission again. The additional information collected during follow‐up and incorporated into our longitudinal discriminant analysis model enables more accurate predictions of long‐term outcome to be made than by simply using baseline information.

## 4 DISCUSSION

We have shown that the longitudinal information collected during follow‐up following a breakthrough seizure, in addition to baseline variables, can be used to identify patients who will not achieve a second period of 12‐month remission. This will be intuitive to clinicians who observe patients during follow‐up and recognize the trajectory of patients likely or unlikely to achieve a seizure remission. In this respect, our model provides further quantitative evidence alongside clinical intuition to support decision‐making.

We have identified that whether a patient has seizures and the number they have between clinic visits are useful indicators of whether they will ultimately achieve a second period of 12‐month remission. In addition, a patient's age at the breakthrough seizure, the number of treatments required to achieve their first period of 12‐month remission, and whether a patient has a neurological insult all have an impact on the likelihood and frequency of seizures experienced following a breakthrough seizure, even if a patient will ultimately achieve a second period of 12‐month remission. In this analysis, we only considered the number of treatments required, rather than the specific treatments, because the number of treatment combinations after withdrawal of randomized drug is too large to model accurately, and differences between the treatments can be small.

Of the patients classified, our model correctly identifies 73% of patients who will not achieve a second period of remission and 84% of those who will. On average, our model classifies those not likely to achieve a second remission after approximately 10 months of follow‐up. Compared to a Cox model, which uses only data up to the time of the breakthrough seizure, our new model is considerably more accurate. This suggests that there is insufficient evidence to predict with accuracy which patients will reachieve remission on the day of the breakthrough seizure and only by observing patients over time can increased confidence be gained. Although this necessitates a delay in being confident about which patients will not achieve remission, we observed that only approximately 10 months of further observation were required.

### 4.1 Limitations

Because we required patients to have experienced a 12‐month remission and a breakthrough seizure with an additional 2 years of follow‐up postseizure, the sample size for our analysis is relatively small. These factors potentially limit the power of our analysis. With larger patient groups, additional baseline covariates relevant in the longitudinal evolution of a patient's seizure history may have been identified. In addition, further longitudinal makers could have been seen to be important predictors of whether a patient will reachieve remission following a breakthrough seizure.

Hughes et al^12^ used the SANAD data to develop a model for identifying patients who will not achieve a 12‐month remission within 5 years of an initial diagnosis. The shorter time frame in this analysis was due to the small number of patients with sufficient follow‐up observations following a breakthrough seizure to consider a longer period (the median follow‐up time postbreakthrough for the 536 patients who experienced a breakthrough seizure was 1.6 years, interquartile range = 0.79‐2.57 years).

With longer follow‐up, a time frame longer than 2 years could have been considered. Many of the patients who did not achieve remission within 2 years may have done so if they had been observed for longer. With a longer period of follow‐up, better predictive accuracy may be achieved.

There is a potential bias in our findings because patients with early first remissions are more likely to be included than patients who achieved a first remission after a longer follow‐up, due to the need for a further 2 years of observation for the patient to be included in our analysis. Similarly, patients who experience a breakthrough seizure closer to their initial remission are more likely to be included than patients who remained in an initial state of remission for longer. Our analysis omitted 210 patients who experienced a breakthrough seizure but were not observed for a further 2 years largely due to the end of the SANAD trial. Because we could not determine the status of these patients, we could not include them in the analysis. Studies with much longer follow‐up would be able to assess this bias.

We excluded patients whose AED dose had been decreased before a breakthrough seizure was experienced. This was because the breakthrough seizure may have been due to drug withdrawal. The predictive tool presented in this paper may not be applicable then to people who have experienced breakthrough seizures following a decrease in AED dose.

For the model presented in this paper to be useful in clinical practice, external validation is required. However, there are no datasets available with the relevant information required to validate our model currently. Internal validation of our model suggests good classification performance.

The SANAD data rely on patient‐reported seizure counts. It may be the case that these are underreported or, in the case of experiencing many seizures, approximated. This may result in our estimates being an underestimation of the actual numbers of seizures experienced, which may in turn bias downward the estimates of the evolution of total seizure counts over time. If patients who do not achieve a second 12‐month remission are expected to experience more seizures, then more accurate seizure counts would increase the separation between the two groups and lead to more accurate classification.

## 5 CONCLUSION

Hughes et al^12^ developed a longitudinal discriminant analysis model to predict patients who will not achieve a first period of 12‐month remission within 5 years of initial diagnosis (sensitivity = 95%, specificity = 97%, AUC = 95% for classified patients). In this paper, we go a step further by developing a discriminant analysis model to identify patients who, having initially achieved 12‐month remission and gone on to have a breakthrough seizure, will not achieve another period of 12‐month remission within 2 years of the breakthrough seizure. We believe that the prediction accuracy of this model is reasonably good, suggesting the potential of this approach to identify patients who will develop drug resistance following an initial remission, although a larger patient sample and longer follow‐up would be required to explore this further.

The predictive model developed here and the one of Hughes et al^12^ provide a useful tool to identify patients who are not likely to achieve remission from seizures early in their clinical follow‐up, both after diagnosis and after a breakthrough seizure following remission. Incorporating these models into an easy to use calculator (possibly as a webtool or app) would be a necessary next step in making these models clinically useful. Such an approach has the potential to provide clinicians with more accurate information regarding the long‐term outcome of their patients, which could lead to improved patient counseling and more informed treatment decisions, including the possibility of aiming for a most tolerated AED regimen rather than total seizure freedom if this was deemed to be unlikely.

## DISCLOSURE

None of the authors has any conflict of interest to disclose. We confirm that we have read the Journal's position on issues involved in ethical publication and affirm that this report is consistent with those guidelines.
